# Effects of *Lactobacillus johnsonii* and *Lactobacillus reuteri* on gut barrier function and heat shock proteins in intestinal porcine epithelial cells

**DOI:** 10.14814/phy2.12355

**Published:** 2015-04-06

**Authors:** Hao-Yu Liu, Stefan Roos, Hans Jonsson, David Ahl, Johan Dicksved, Jan Erik Lindberg, Torbjörn Lundh

**Affiliations:** 1 Department of Animal Nutrition and Management, Swedish University of Agricultural SciencesUppsala, Sweden; 2 Department of Medical Cell Biology, Uppsala UniversityUppsala, Sweden; 3 Department of Microbiology, Uppsala BioCenter, Swedish University of Agricultural SciencesUppsala, Sweden

**Keywords:** Cytoprotective heat shock protein 27 and 72, intestinal barrier integrity, IPEC-J2 cell line, probiotic lactobacilli, tight junction protein

## Abstract

Heat shock proteins (HSPs) are a set of highly conserved proteins that can serve as intestinal gate keepers in gut homeostasis. Here, effects of a probiotic, *Lactobacillus rhamnosus* GG (LGG), and two novel porcine isolates, *Lactobacillus johnsonii* strain P47-HY and *Lactobacillus reuteri* strain P43-HUV, on cytoprotective HSP expression and gut barrier function, were investigated in a porcine IPEC-J2 intestinal epithelial cell line model. The IPEC-J2 cells polarized on a permeable filter exhibited villus-like cell phenotype with development of apical microvilli. Western blot analysis detected HSP expression in IPEC-J2 and revealed that *L. johnsonii* and *L. reuteri* strains were able to significantly induce HSP27, despite high basal expression in IPEC-J2, whereas LGG did not. For HSP72, only the supernatant of *L. reuteri* induced the expression, which was comparable to the heat shock treatment, which indicated that HSP72 expression was more stimulus specific. The protective effect of lactobacilli was further studied in IPEC-J2 under an enterotoxigenic *Escherichia coli* (ETEC) challenge. ETEC caused intestinal barrier destruction, as reflected by loss of cell–cell contact, reduced IPEC-J2 cell viability and transepithelial electrical resistance, and disruption of tight junction protein zonula occludens-1. In contrast, the *L. reuteri* treatment substantially counteracted these detrimental effects and preserved the barrier function. *L. johnsonii* and LGG also achieved barrier protection, partly by directly inhibiting ETEC attachment. Together, the results indicate that specific strains of *Lactobacillus* can enhance gut barrier function through cytoprotective HSP induction and fortify the cell protection against ETEC challenge through tight junction protein modulation and direct interaction with pathogens.

## Introduction

Early control of intestinal disorders in newborns is crucial as they are highly susceptible to pathogens, such as enterotoxigenic *Escherichia coli* (ETEC). The consequent infection can be fatal for young animals, especially postweaning piglets and children under the age of five (Bailey 2009; Croxen and Finlay 2010). The pathogenesis of ETEC starts with bacterial attachment to the host small intestinal epithelium cells (IECs), followed by production of heat-labile and heat-stable enterotoxins. These toxins facilitate more intimate pathogen colonization, disrupt the tight junction (TJ) structure of the mucosal barrier, and result in a ‘leaky’ gut. This is followed by pathogen internalization, where the pathogen subverts host cell processes and manipulates pathways in coordination with invasion, ultimately leading to cell death (Handl et al. 1988; Croxen and Finlay 2010). In the face of pathogen challenge, it is essential to constitute an efficient intestinal barrier that separates the internal tissue from the external environment to provide the front line of defense.

The maintenance of barrier function is associated with dynamic modulation of the TJ complex, which encloses IECs against the uptake of food antigens, gut microbes, and other macromolecules (Ulluwishewa et al. 2011). In this regard, the porcine jejunal epithelial cell line IPEC-J2 is a suitable in vitro model for investigating interactions between bacteria (commensal or transient) and the small intestinal epithelium. IPEC-J2 provides high specificity for pig studies and is analogous to human gut physiology (Brosnahan and Brown 2012). IPEC-J2 cells grown on permeable filters allow epithelium differentiation and polarization in a two-compartment system (apical and basolateral), therefore reconstituting a small intestinal villus-like cell phenotype (Geens and Niewold 2011; Diesing et al. 2012; Zakrzewski et al. 2013).

A substantial body of evidence suggests that probiotics are able to counteract the pathogenic effects of ETEC (Guarner 2008; Ringel et al. 2012; Klingspor et al. 2014). In particular, lactobacilli, normal inhabitants of the small intestine, are commonly used as probiotics in human and animal applications (De Lange et al. 2010; Ringel et al. 2012). Earlier studies have shown promising effects of probiotic lactobacilli. For instance, *Lactobacillus rhamnosus* GG (LGG) protects intestinal Caco-2 cells from ETEC K88-associated inflammation (Roselli et al. 2006) and young adult mouse colon cells (YAMC) from oxidant stress (Tao et al. 2006). Certain strains of *Lactobacillus reuteri* can ameliorate dextran sodium sulfate-induced colitis in rats, where the protective mechanism seems to be associated in maintaining intestinal barrier integrity (Dicksved et al. 2012). Furthermore, it has been shown that *Lactobacillus johnsonii* strain JCM 2012^T^ is involved in the regulation of IL-12 production, which influences host homeostasis (Shida et al. 2009). Indeed, probiotics exhibit a great diversity of functions and the mechanisms of importance in promoting health need further elucidation.

Induction of cytoprotective heat shock protein (HSP) 27 and HSP72 in IECs is one action that can be taken by probiotics in sustaining intestinal homeostasis (Petrof et al. 2004; Liu et al. 2014b). The resulting HSP carries out crucial housekeeping functions to maintain mucosal barrier integrity against various stimuli in the intestinal microenvironment. HSP27 is associated with cytoskeleton stabilization (Mounier and Arrigo 2002), whereas both HSP27 and HSP72 (homolog to HSP70) display chaperone properties, ranging from folding peptides into advanced structures, refolding, and restoring damaged proteins in order to deliver them to proper locations and confer cell protection (Kampinga and Craig 2010).

Exogenous HSP27 has been observed to stimulate overproduction of IL-10 (a major anti-inflammatory cytokine) in human monocytes, indicating that HSP27 may be secreted extracellularly and may play an important role in immune system modulation (De et al. 2000). Moreover, HSP70 has been shown to induce IL-10 production, which indicates a possible immunomodulatory role of HSPs in host health and the development of novel therapeutic strategies (Borges et al. 2012). We have previously demonstrated that *Lactobacillus* spp. (mainly *L. johnsonii and L. reuteri* species) dominate the porcine small intestine and are promoted by dietary inclusion of chicory pectin and prebiotic inulin (Liu et al. 2012a). Furthermore, we have shown that the relative abundance of *Lactobacillus* spp. is strongly correlated with expression of HSP72 in the small intestine of young pigs. In addition, we have observed induction of HSP27 in Peyer's patches, which contain high numbers of immune cells (Liu et al. 2014a).

Therefore, we isolated lactobacilli from pigs with high mucosal expression of HSP27 and HSP72 in order to study how specific strains of lactobacilli and their metabolites could influence gut barrier function and HSP expression in an IPEC-J2 cell model. Our hypothesis was that different *Lactobacillus* species possess the ability to maintain the intestinal barrier function under ETEC challenge in different ways and that the mechanism behind this protective effect could be dependent on the ability to modulate expression of HSPs or TJ proteins, or to restrict adhesion of pathogens.

## Material and Methods

### Epithelial cell line and culture condition

The porcine jejunal epithelial IPEC-J2 cell line was used throughout the experiment (kindly provided by Nguyen Lien Thi Minh and Kerstin Skovgaard, Technical University of Denmark, National Veterinary Institute, Denmark). IPEC-J2 is a nontransformed small intestinal cell line developed from a neonatal, unsuckled piglet (<1 day old), maintained as a culture (Berschneider 1989) and characterized as a swine-specific in vitro infection model (Schierack et al. 2006). The cells were grown in Dulbecco's modified Eagle's medium/F-12 Ham containing 0.12% sodium bicarbonate, 15 mM HEPES, pyridoxine and l-glutamine (Sigma Aldrich, St. Louis, Missouri), supplemented with an antibiotic mixture (penicillin, 100 U/mL, and streptomycin, 100 *μ*g/mL), 0.5 mmol/L sodium pyruvate, and 5% fetal bovine serum (Sigma Aldrich), and maintained in an atmosphere of 5% CO_2_ at 37°C. Cells were passaged every 3–4 days (by seeding at 1:3 ratio). Medium was changed every other day. During experimental periods with bacterial incubation, cell culture medium was replaced with medium containing no antibiotics at least 12 h prior to treatment. In order to keep the cell phenotype stable and consistent (Hubatsch et al. 2007), cells within 10 passages (passage number 95–105) were used in all experiments.

### Transepithelial electrical resistance and scanning electron microscopy

IPEC-J2 cells were seeded on Transwell®-COL collagen-coated membrane filters (1.12 cm^2^; 0.4 *μ*m; Corning, New York) to allow monolayer polarization. IPEC-J2 cells were seeded at 5 × 10^5^ cells per filter on transwell membranes in 12-well tissue culture plates. Transepithelial electrical resistance (TEER) was measured every day after seeding using the Millicell Electrical resistance system (Millipore, Darmstadt, Germany). The high seeding density was to saturate the available area in order to avoid cell division (Geens and Niewold 2011). At each measurement, duplicate measurements from two different areas of the filter were performed and the results were expressed as Ω cm^2^. The cell monolayer with TEER above 4000 Ω cm^2^ was assumed to be polarized (Geens and Niewold 2011; Diesing et al. 2012). Samples with TEER under 1000 Ω cm^2^ before bacterial treatment were discarded. Furthermore, scanning electron microscopy analysis was performed on day 9 after seeding in order to confirm that the IPEC-J2 monolayer cells were polarized. Transwell filters with untreated cells were fixed with 2.5% glutaraldehyde (in distilled water) for 2 h at room temperature (RT), followed by five washing with distilled water (10 min each time). Then, 2% osmium tetroxide was applied to the filters for 2 h (RT), followed by washing in distilled water as described above. Dehydration was performed using graded levels of ethanol, at 25%, 50%, 70%, and 95%, for 10 min each (RT) per ethanol level, followed by two rinses in 100% ethanol (10 min, RT). The analysis was carried out using a Hitachi TM-1000-*μ*DeX environmental tabletop electron microscope. The microanalysis data were processed by MicroKemi AB (Uppsala, Sweden) and each assay was comprised of at least three independent experiments.

### Bacterial strain selection and preparation

The bacterial strains used in the study are described in Table1. ETEC strain 853/67 is a clinical isolate from pigs that has been reported to test positive for at least three kinds of enterotoxins (heat-labile enterotoxin and heat-stable enterotoxins I and II; Handl et al. 1988). Three strains of lactobacilli were used: LGG (ATCC 53103), *L. johnsonii* P47-HY, and *L. reuteri* P43-HUV. The latter two were isolated from ileal digesta from healthy pigs (Liu et al. 2012a) by cultivation on de Man-Rogosa-Sharp (MRS) agar (Oxoid, U.K.) at 37°C for 72 h in an anaerobic atmosphere. In total, 12 bacterial colonies with varying morphology were selected and identified by 16S rRNA gene sequence determination of PCR products generated with the general primers Bact-8F (5′-AGAGTTTGATCCTGGCTCAG-3′), and 926r (5′-CCGTCAATTCCTTTRAGTTT-3′). Consequently, two strains (*L. johnsonii* P47-HY and *L. reuteri* P43-HUV) among the 12 isolates were chosen for further studies in the cell model. The 16S rRNA gene sequences for these isolates were deposited in GenBank at NCBI under the accession numbers KF267448 and KF267449. Moreover, the gene encoding propanediol dehydratase large subunit (*pduC*) was detected by PCR analysis in the *L. reuteri* strain P43-HUV, indicating that this strain can produce the antimicrobial compound reuterin (Walter et al. 2011). All bacteria cultures were freshly prepared for each experiment.

**Table 1:** Bacterial strains used in the study

Strain	Species	Description	Source
GG	*Lactobacillus rhamnosus*	Healthy human intestine, pH of supernatant 4.00	Probiotic product Culturelle ATCC 53103
P47-HY	*Lactobacillus johnsonii*	Healthy pig small intestine, pH of supernatant 4.69	This study
P43-HUV*	*Lactobacillus reuteri*	Healthy pig small intestine, pH of supernatant 4.91	This study
853/67	Enterotoxigenic *Escherichia coli*	Porcine clinical isolate/positive for phenotype LT, ST_I_, ST_II_, and K88+	Handl et al. (1988)

* The gene encoding propanediol dehydratase large subunit (*pduC*) was detected by PCR analysis; LT, heat-labile enterotoxin; ST, heat-stable enterotoxin.

ETEC was grown in Luria-Bertani broth (tryptone 10 g; yeast extract 5 g; NaCl 5 g/L distilled water) at 37°C overnight, with vigorous shaking at 120 rpm. *Lactobacillus* spp. was grown in MRS broth at 37°C overnight. The optical density (*Lactobacillus* spp. OD_595_ ≈ 8.4; ETEC OD_595_ ≈ 6.4) of each bacterial strain was measured by a cell density meter (Nordic Biolabs, Täby, Sweden). Bacterial numbers were counted using a hemocytometer under light microscope based on serial dilutions. For each experiment, bacteria were harvested by centrifugation at 5000 × g at 4°C for 10 min and washed with PBS (pH 7.4). The bacterial pellet was resuspended in prewarmed, antibiotic-free cell culture medium to the desired concentration, while the supernatant was collected as previously described (Tao et al. 2006). In brief, the supernatant from each bacterium (sLGG, sLj, and sLr) was filtered through a 0.22-*μ*m filter (Sarstedt, Nümbrecht, Germany) to remove remaining bacterial cells. Aliquots of each supernatant were stored at −80°C until further use. Prior to treatment of the IPEC-J2 cells, supernatant was diluted (1:10 directly into antibiotic-free cell culture medium). The pH values of the solutions before and after dilution were determined using a common pH-meter (PHM 210, Radiometer, Villeurbanne Cedex, France). In this study, supernatant and bacteria from the early stationary growth phase were used.

### Cell viability test

The viability (membrane integrity) of cells treated with live bacteria was assessed by a lactate dehydrogenase (LDH) assay according to the manufacturer's protocol using 25 *μ*L cell culture media (BioVision, Milpitas, CA). In brief, IPEC-J2 cells were seeded at 6 × 10^4^ cells per well in 96-well tissue culture plates and grown for 24 h. Medium and nonadherent cells were then removed and washed once with PBS. Cells were untreated or treated with bacteria (prepared as described above) at different concentrations representing multiplicity of infection (MOI) of 100 for lactobacilli and 10 for ETEC, and incubated at 37°C in a 5% CO_2_, 95% air-humidified incubator for 12 h. Various MOI for different bacterial species (1000 or 100 for lactobacilli and 100 or 10 for ETEC) and time of incubation (2, 6, and 24 h) were studied in preliminary experiments. The MOI and time of incubation that allowed bacterial–epithelium interaction and partial cell damage without complete disruption of the cell monolayer due to bacteria overload were chosen. The cell viability was further confirmed by direct cell counting after detachment by trypsin-EDTA (Sigma Aldrich). The cells were enumerated with a hemocytometer under light microscope with only live cells taken into account, that is, excluded Trypan blue stained cells.

### Bacterial adhesion assay

Adhesion of the three *Lactobacillus* strains and their ability to inhibit ETEC adhesion were tested on IPEC-J2 cell monolayer by the agar plating method described by Roselli et al. (2007). In brief, IPEC-J2 cells were seeded on 24-well tissue culture plates at 5 × 10^5^ cells per well. After 24 h of growth, the cells were treated with LGG, *L. johnsonii*, *L. reuteri* (MOI of 100), and ETEC (MOI of 10) alone, or simultaneously with ETEC and lactobacilli for 2 h to allow bacterial adhesion. To determine whether *Lactobacillus* metabolites could inhibit ETEC adhesion, the supernatants (sLGG, sLj, and sLr) were added to the cells containing ETEC (MOI 10). After incubation, nonadherent bacteria were removed from the cell cultures by washing the wells three times with PBS. IPEC-J2 cells were lysed with 1% Triton X-100 and adhered bacteria were quantified by plating serial dilutions of the cell culture lysate on MRS agar for lactobacilli or Luria-Bertani agar for ETEC. Control experiments showed that the lactobacilli were not able to grow on Luria-Bertani plates (ETEC-specific) at 37°C overnight under aerobic conditions.

### Western blot analysis of heat shock proteins

For HSP determination, IPEC-J2 cells were seeded at 1.5 × 10^6^ cells per well in six-well tissue culture plates and grown to 100% confluence (around 24 h). It has been shown that supernatant from LGG induces HSP expression in YAMC cells after 4–6 h of incubation (Tao et al. 2006). In the present study, cells were untreated or treated with viable LGG, *L. johnsonii*, and *L. reuteri* (MOI of 100) or their equivalent supernatant (sLGG, sLj, and sLr) for 6 h, then washed once with PBS and twice with antibiotic-free cell culture medium (prewarmed) and placed back in the incubator for 6 h. The cells were collected in ice-cold PBS.

Proteins were extracted from samples using a mammalian cell extraction kit (BioVision) and cell lysates were stored at −80°C for further analysis. One aliquot of sample was used for protein determination by the bicinchoninic acid assay (Pierce, Rockford, IL). For electrophoresis, samples were boiled at 95°C for 1 min after addition of sodium dodecyl sulfate-2-mercaptoethanol buffer (SDS-ME). A sample of 2.5–4 *μ*L, equivalent to 4 *μ*g of protein was loaded on the gel and was then separated by 8–25% gradient SDS-PAGE with PhastSystem (GE Healthcare, Uppsala, Sweden) and transferred to a nitrocellulose membrane. Membranes were blocked in 5% (wt/vol) skim milk in PBS containing 0.05% (vol/vol) Tween 20 (PBS-T) for 1 h at RT and then incubated with agitation overnight at 4°C with anti-HSP27 and anti-HSP72. To verify equal loading of samples, the membranes was incubated with anti-heat shock cognate protein (HSC)73 antibody (in PBS-T, Stressgen, Sandiego, CA). In all experiments, heat shock controls used were cells that were heat treated at 42°C for 60 min and left at 37°C for 2 h before harvest, based on preliminary experiments and a previous study (Ren et al. 2001). The nitrocellulose membranes containing blotted proteins were washed five times in PBS-T for 10 min at RT, then incubated for 1 h with species-appropriate horseradish peroxidase-conjugated secondary antibody (in PBS-T; Enzo Life Sciences, Solna, Sweden), washed five times in PBS-T for 10 min, followed by a final wash in PBS (no Tween), and developed using diaminobenzidine solution (VectorLAB, Burlingame, CA). The densitometry was performed using a computer-assisted image analysis system (Quantity One, version 4.6.7; Bio-Rad, Hercules, CA) under the same conditions in all experiments, normalized to the intensity of HSC73 for equal protein loading control as described previously (Segawa et al. 2011; Paszti-Gere et al. 2012).

### Localization of ZO-1

Cells were grown on transwell filters in 12-well tissue culture plates as described above. On day 9, the monolayer reaching polarization was untreated or apically treated with lactobacilli (MOI 100) for 6 h. Thereafter, each well was vigorously washed with medium three times to eliminate stimulants and then challenged with ETEC (MOI 10) for 3 h. Different time of incubation were chosen based on cell viability test and HSP detection in IPEC-J2 cell line as described above. Filters were fixed in 4% paraformaldehyde (4°C) for 15 min at 4°C and permeabilized with 0.2% Triton-X-100 for 15 min at RT. Membranes were rinsed with PBS and detached from the transwell. The detached membrane with cells was then incubated in a 3% BSA solution (Sigma Aldrich) for 1 h at RT. Rabbit polyclonal anti-ZO-1 (diluted at 1:100; N-term; Invitrogen, Carlsbad, CA) was applied as primary antibody and Alexa fluor 488 goat anti-rabbit (green) as secondary antibody (diluted at 1:200; Invitrogen). Nuclei were stained with DAPI (Invitrogen). Images were acquired using laser scanning confocal microscopy (Nikon C-1 with Plan ApoVC 60×/1.40 oil objective; Nikon EZ-C1 software; Nikon, Amsterdam, the Netherlands).

### Statistical analysis

All the assays were performed in at least three independent experiments. One way ANOVA followed by Tukey's post hoc test was used to test treatment effects using SAS package 9.2 (SAS Institution, Cary, NC). Differences were considered significant at *P *<* *0.05.

## Results

**Figure 1:**
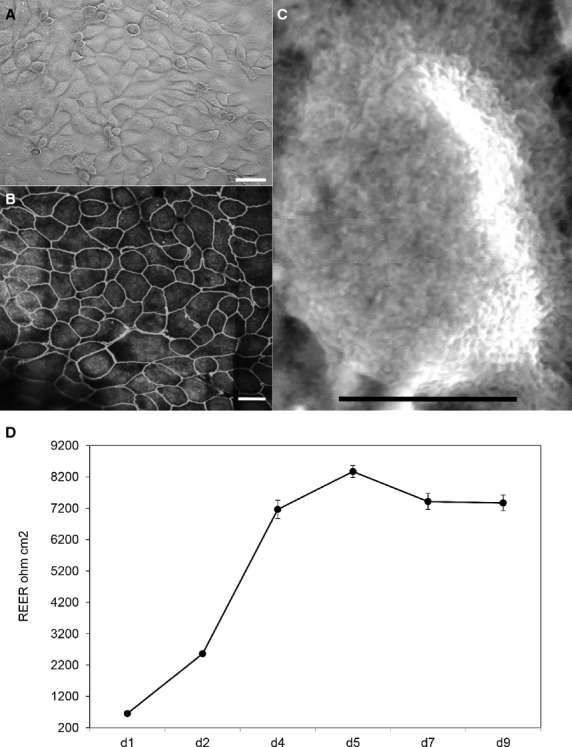
Characterization of IPEC-J2 cell monolayer. (A) Light microscope image of confluent cell monolayer grown on a tissue culture flask, scale bar equals 20 *μ*m. (B) Confocal micrograph showing a top view of IPEC-J2 monolayer grown on transwell filter for 9 days, the cell borders can be distinguished by immunofluorescent staining of tight junction protein ZO-1, scale bar equals 20 *μ*m. (C) Top view of well-differentiated IPEC-J2 cell with microvilli observed by scanning electron microscopy, figure contains one cell from cell monolayer grown on transwell filter for 9 days, scale bar equals 5 *μ*m. (D) Progression in transepithelial electrical resistance (TEER) values of cells grown on transwell filter for 9 days. Data are given as means (±SEM) of 20 separate experiments. They were assigned to experimental treatment on day 9, respectively.

The IPEC-J2 cells were seeded at approximately 1 × 10^5^ cell/mL in T25 flasks and reached confluence after 3 days (Fig.1A). IPEC-J2 cells cultured on transwell filter for 9 days developed TJs (Fig.1B) and microvilli (Fig.1C). Intestinal barrier integrity measured by TEER increased dramatically from day 2 to day 5 and then plateaued (Fig.1D). The TEER value did not decrease to 1000 Ω cm^2^ until day 21 (data not shown).

### Effect of *Lactobacillus* spp. and ETEC on IPEC-J2 cell viability

**Figure 2:**
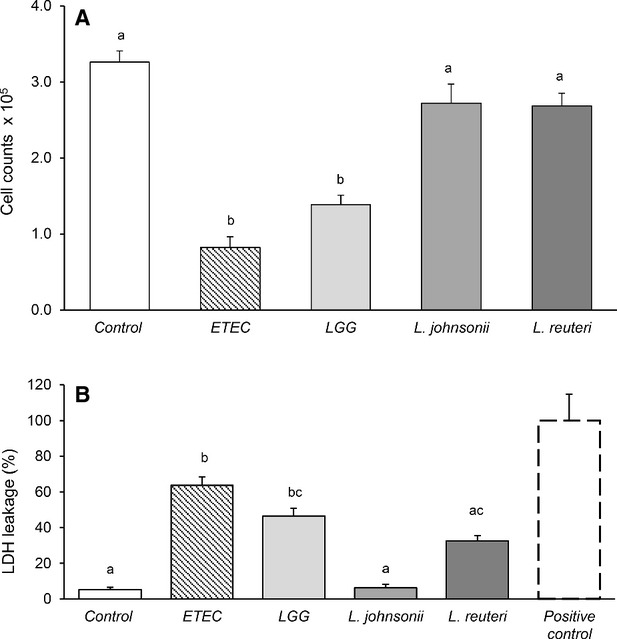
Effect of *Lactobacillus* spp. and ETEC on IPEC-J2 cell viability. Cells grown on 96-well tissue culture plates and reached a confluent layer were left untreated or treated with bacterial cells of LGG, *L. johnsonii*, and *L. reuteri* at multiplicity of infection (MOI) of 100 and ETEC (MOI 10) for 12 h. (A) Live IPEC-J2 cell counts. (B) Percentage of IPEC-J2 cell lactate dehydrogenase (LDH) activity relative to the average LDH value of positive control cells (whole cell lysate). Data are given as means (±SEM) of four separate experiments. Values with different letters differ significantly (*P *<* *0.0001).

Twelve hours of incubation with bacteria showed that the survival of IPEC-J2 cells was partly affected, an effect most likely caused by bacterial growth. The number of live cells was not different after *L. johnsonii* and *L. reuteri* treatment compared with the untreated control, whereas ETEC and LGG significantly decreased the cell viability (Fig.2A). A similar response was observed on LDH activity, calculated as percentage of positive control (higher LDH activity indicates more cell membrane leakage; Fig.2B). The relative LDH activity was lowest in the control group and *L. johnsonii* treatment, followed by *L. reuteri-* and LGG-treated cells, whereas ETEC exhibited the highest relative LDH activity compared to the other groups (*P *<* *0.0001). However, a 6-h incubation with LGG did not affect LDH release compared to the control (relative LDH activity of untreated control group, 8.8 ± 1.49% and from LGG alone treatment, 14.9 ± 5.09%; *P *>* *0.05, *n* = 3).

### Adherence of *Lactobacillus* spp. and ETEC to IPEC-J2 cells

**Figure 3:**
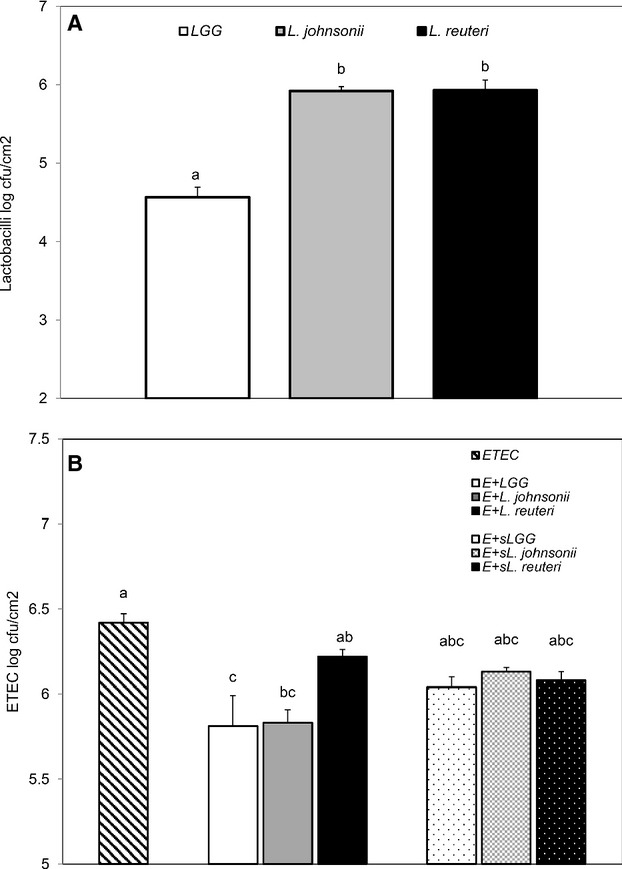
Adhesion of bacteria on IPEC-J2 cells. Cells grown on 24-well tissue culture plates were treated with bacterial cells of LGG, *Lactobacillus johnsonii*, and *Lactobacillus reuteri* (multiplicity of infection, MOI 100) or ETEC (MOI 10) alone, or with coincubations of ETEC (MOI 10) and *Lactobacillus* spp. (MOI 100) or their equivalent supernatants for 2 h. (A) Adhesion of LGG, *L. johnsonii*, and *L. reuteri*. (B) Reduction in ETEC adhesion by *Lactobacillus* spp. and their equivalent supernatants sLGG, s*L. johnsonii,* and s*L*. *reuteri*. The values given are means (±SEM) of four separate experiments. Values with different letters differ significantly (*P *<* *0.001).

LGG, *L. johnsonii*, and *L. reuteri* were all able to adhere to IPEC-J2 cells after 2-h incubation. *L. johnsonii* and *L. reuteri* showed similar adhesion ability, whereas adhesion was significantly lower for LGG (Fig.3A). Co-incubation of ETEC with either LGG or *L. johnsonii* strongly inhibited the attachment of ETEC to IPEC-J2 cells. In contrast, co-incubation with *L*. *reuteri* and ETEC did not reduce ETEC attachment (Fig.3B). Furthermore, the supernatants sLGG, sLj, and sLr did not exclude ETEC adhesion (Fig.3B).

### Effects of *Lactobacillus* spp. on HSP27 and HSP72 expression in IPEC-J2 cells

**Figure 4:**
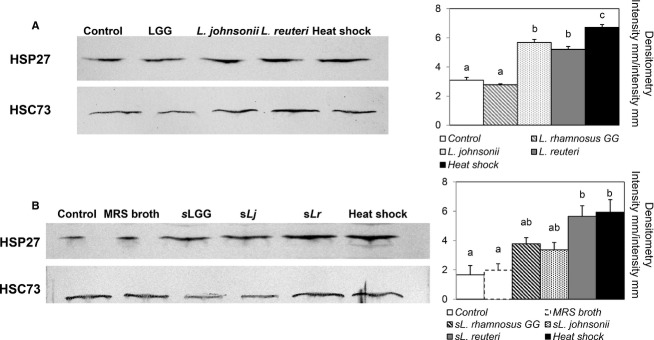
Effect of lactobacilli on heat shock protein (HSP) 27 expression in porcine IPEC-J2 epithelial cells. (A) Cells grown on six-well tissue culture plates and reached a confluent layer were left untreated or treated with bacterial cells of LGG, *Lactobacillus johnsonii*, and *Lactobacillus reuteri* at multiplicity of infection (MOI) of 100 for 6 h, followed by a 6-h recovery. (B) Results from stimulation with supernatants from LGG, *L. johnsonii*, and *L. reuteri* (sLGG, sLj, and sLr). MRS broth was used as a vehicle control. In all experiments, the heat shock treatment, where cells were heat treated for 60 min at 42°C followed by a 2-h recovery, served as a positive control. Densitometry of HSP27 expression was calculated using heat shock cognate protein (HSC) 73 as equal protein loading control. Images shown represent three separate experiments. Data given are means (±SEM). Values with different letters differ significantly (*P *<* *0.001).

**Figure 5:**
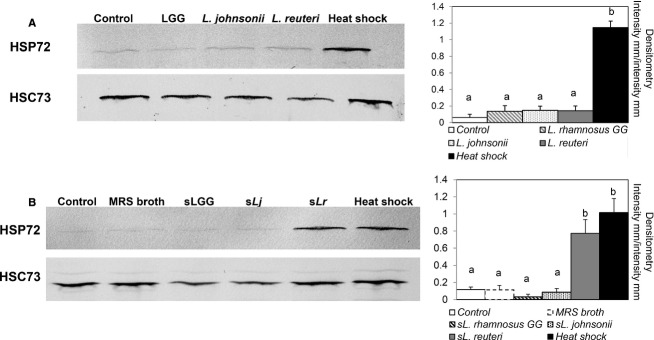
Effect of lactobacilli on heat shock protein (HSP) 72 expression in porcine IPEC-J2 epithelial cells. (A) Cells grown on six-well tissue culture plates and reached a confluent layer were left untreated or treated with bacterial cells of LGG, *Lactobacillus johnsonii*, and *Lactobacillus reuteri* at multiplicity of infection (MOI) of 100 for 6 h, followed by a 6-h recovery. (B) Results from stimulation with supernatants from LGG, *L. johnsonii*, and *L. reuteri* (sLGG, sLj, and sLr). MRS broth was used as a vehicle control. In all experiments, the heat shock treatment, where cells were heat treated for 60 min at 42°C followed by a 2-h recovery, served as a positive control. Densitometry of HSP72 expression was calculated using heat shock cognate protein (HSC) 73 as equal protein loading control. Images shown represent three separate experiments. Data are given as means (±SEM). Values with different letters differ significantly (*P *<* *0.001).

Research is limited regarding intestinal HSP expression in pigs (David et al. 2002). To our knowledge, this is the first study on HSP27 and HSP72 expression in the IPEC-J2 cell line stimulated with porcine-specific bacteria. The HSP-inducing effects of the different strains of lactobacilli and their supernatants are shown in Figures4 and 5. The constitutively expressed HSC73 did not change in response to treatment, indicating that the expression of HSP27 and HSP72 was specifically induced by bacterial stimulation. HSP27 was expressed in all samples and showed a high basal level, including in the control group. The most robust response with live bacteria treatment was obtained with *L. johnsonii* and *L. reuteri* (Fig.4A). In contrast, LGG did not provoke stronger HSP27 expression compared with the control. Similar responses were observed with the supernatant treatments, which stimulated expression of HSP27 more strongly compared with the stimulation from live bacteria (Fig.4B). Supernatant from *L. reuteri* strongly increased HSP27 expression in IPEC-J2 cells, and reached the same level as the heat shock control.

Unlike the overall strong expression of HSP27 in IPEC-J2 cells, 6 h of incubation with bacteria only provided detectable levels of HSP72 and the expression was similar regardless of strain (Fig.5A). However, the supernatant of *L. reuteri* did induce strong HSP72 expression (Fig.5B).

### Protection by *Lactobacillus* spp. against ETEC challenge

**Figure 6:**
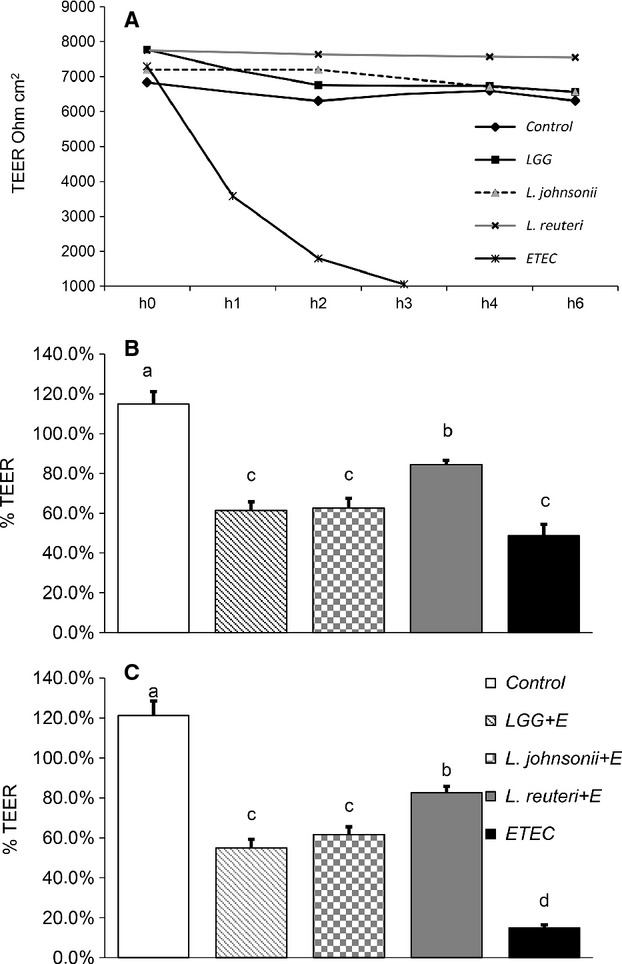
Effects of bacteria on transepithelial electrical resistance (TEER) in IPEC-J2 cells grown on transwell filters. (A) Polarized cells left untreated or treated with bacterial cells of LGG, *Lactobacillus johnsonii*, and *Lactobacillus reuteri* at multiplicity of infection (MOI) of 100 for 6 h or ETEC (MOI 10) for 3 h. (B) First hour in which IPEC-J2 cells were challenged by ETEC (MOI 10) after being preincubated with *Lactobacillus* spp. (C) Last hour (hour 3) in which IPEC-J2 cells were challenged by ETEC (MOI 10) after being preincubated with *Lactobacillus* spp. TEER values are expressed in Ω cm^2^. The controls showing higher values than 100% because the percentage of TEER changes during incubation with bacteria was calculated based on the TEER values of control cells at h 0. Data given are means (±SEM) of at least four separate experiments. Values with different letters differ significantly (*P *<* *0.001).

Our starting hypothesis was that treatment of ETEC at MOI 10 to polarized IPEC-J2 cell monolayer disrupts barrier integrity and destroys TJs. TEER was used as an indicator of the intestinal barrier integrity (Cario et al. 2004; Klingberg et al. 2005) (the percentage of TEER changes during incubation with bacteria was calculated based on the TEER values of control cells at h 0). In control cells, TEER was constant throughout the experimental period, whereas in ETEC-infected IPEC-J2 cells the TEER value decreased in 1 h of incubation and reached 1000 Ω cm^2^ at 3 h, indicating a significant compromise of the cell monolayer in the current study (Fig6A). TEER values decreased in the first hour with preincubation of lactobacilli followed by ETEC challenge in all groups compared with the control (Fig.6B). However, compared with ETEC treatment alone, TEER in *L. reuteri* pretreated cells was retained. The 3-h incubation with ETEC did not change the TEER value for *Lactobacillus* pretreated cells and the *L. reuteri* pretreatment continued to have higher TEER values compared with LGG and *L. johnsonii* (Fig.6C). However, the 3-h incubation caused a significant reduction in TEER in cells treated with ETEC alone (Fig.6C).

**Figure 7:**
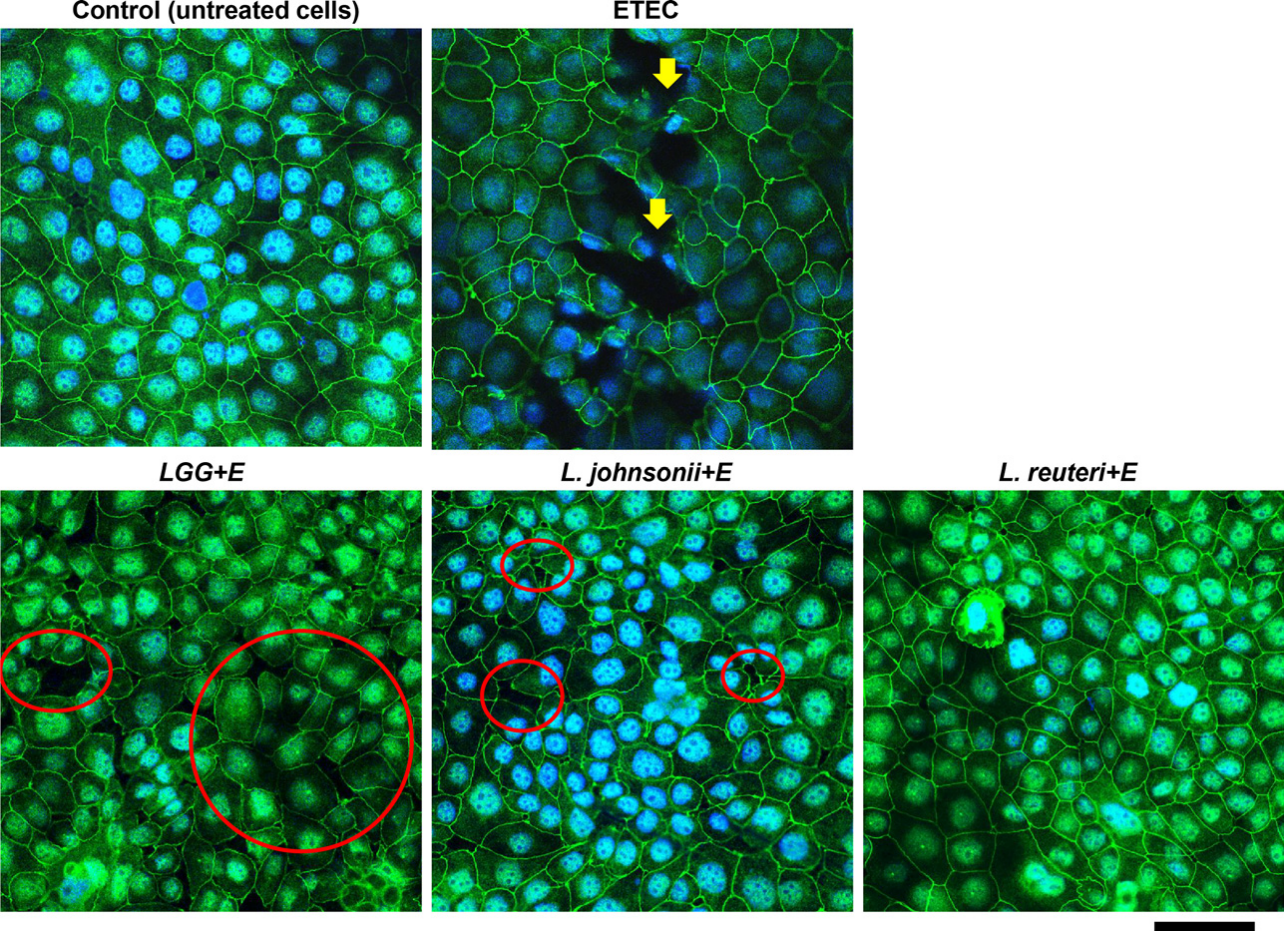
Cellular distribution of the tight junction protein ZO-1 in IPEC-J2 cells. Cell monolayers grown on transwell filters were left untreated (control) or treated with bacterial cells of ETEC (multiplicity of infection, MOI 10) alone for 3 h or pretreated with *Lactobacillus* spp. (MOI 100) for 6 h and then challenged by ETEC for 3 h. Monolayers stained for the tight junction protein ZO-1 (green) and nuclei stained with DAPI (blue) were detected by laser scanning confocal microscopy. The images were representatives from three separate experiments. Yellow arrows show the broken lining of ZO-1 expressions. Red circles highlight areas of cell disassociation. Scale bar equals 50 *μ*m for all images.

To confirm the dramatic effect of ETEC challenge on TEER, immunolocalization of the TJ protein ZO-1 was performed (Fig.7). Uninfected IPEC-J2 cells exhibited uniformly expressed ZO-1 between adjacent cells, with a typical chicken-wire pattern. The ZO-1 was detected as a continuous lining. In comparison, a challenge with ETEC caused different degrees of cell monolayer damage. TJ openings were observed, which shows a loss of cell–cell contact. The visualized ZO-1 lining was weakened and broken by ETEC challenge (Klingspor et al. 2014). Furthermore, in some fields of the monolayer, nuclei staining were diminished, indicating cell death.

We next examined whether the lactobacilli could enhance the cell integrity to resist such challenge. ZO-1 staining revealed that different strains of *Lactobacillus* spp. affected IPEC-J2 cells differently (Fig.7). With preincubation of LGG, TJ openings were observed after ETEC challenge. Cell dissociations and disconnections appeared frequently on the monolayer. In comparison, *L. johnsonii* pretreated cells showed fewer cell dissociations. The monolayer appeared intact, but small TJ openings were seen (Fig.7). In contrast, preincubation with *L. reuteri* completely preserved the cell monolayer from ETEC challenge, with ZO-1 remaining evenly distributed and intact. It is noteworthy that although cell dissociation was present to different degrees, broken lining of ZO-1 expression or cell death was never observed in cells with lactobacilli pretreatment compared with the ETEC treatment alone.

## Discussion

Probiotic lactobacilli can provide benefits to the host gut through a diverse set of mechanisms that include competitive exclusion of pathogens (Roselli et al. 2007), production of antimicrobial compounds (Walter et al. 2011), modulation of host immune responses (Shimazu et al. 2012), and maintenance of intestinal barrier integrity (Karczewski et al. 2010). In the present study, we demonstrated that IPEC-J2 monolayers, preincubated with a novel *L. reuteri* strain isolated from pig, significantly attenuated the ETEC-induced disruption of ZO-1 in the vicinity of TJ structures and the loss of intestinal barrier integrity, whereas *L. johnsonii* and LGG provided less protection under ETEC challenge. *Lactobacillus* spp. have been shown to improve intestinal barrier function and mitigate ETEC infection in IECs (Roselli et al. 2006, 2007; Shimazu et al. 2012), partly by modulating TJ protein expression and distribution (Karczewski et al. 2010; Ulluwishewa et al. 2011). The TJ complex consists of numerous proteins, including the scaffolding protein ZO-1 and the transmembrane proteins occludin and claudins, and plays a pivotal role in maintenance of barrier function and prevention of bacterial infections in the host. A substantial connection between ZO-1 tightening and increasing TEER, eliciting changes in occludin and claudin-1 distribution in adjacent epithelial cells, has been described in human IEC cell lines (Cario et al. 2004). IPEC-J2 cells show much higher TEER than other cell lines, for example CMT-93, HT-29/B6 and (Zakrzewski et al. 2013). The high variation in TEER values between different cell lines can be due to differences in the TJ composition and length, species differences and if the cells are transformed or nontransformed. Even cell culturing factors as which serum (bovine or porcine) is used influence on TEER values (Zakrzewski et al. 2013).

The present study showed that ETEC caused loss of cell–cell contact, reduction in TEER, disruption of ZO-1, and eventually cell death. In contrast, lactobacilli pretreated cells that were challenged with ETEC displayed varied monolayer wholeness, but never the broken linings or cell death indicating pathogen internalization. It is conceivable that the pretreatment with *Lactobacillus* spp. in our study strengthen the epithelial monolayer integrity, in particular for *L. reuteri*. For *L. johnsonii* and LGG pretreated cells, the opening and dissociation of cell monolayer was seen under challenge to differing degrees.

Several studies have demonstrated that probiotics confer protection in the host gut against various stimuli, partly by induction of cytoprotective HSP27 and HSP72 in the intestinal epithelium (Liu et al. 2014b). It has recently been suggested that induction of HSP could be linked with ZO-1 expression, with reports that mother's milk-induced HSP70 expression is colocalized with ZO-1 expression in rat small intestine, indicating early protection of the immature gut (Liedel et al. 2011). We found that the IPEC-J2 cell line had a generally high expression of HSP27. This confirms results in our earlier study, where HSP27 was expressed in all tissue samples from both the small and large intestine of healthy young pigs, regardless of diet treatment (Liu et al. 2012b). Similar results have been reported in a weaning piglet study (David et al. 2002). In contrast, HSP25/27 (HSP25 is a homolog to HSP27) is reported to be barely expressed in YAMC and human Caco2/bbe cell lines without induction (Kojima et al. 2003; Ueno et al. 2011). This discrepancy could be dependent on the cell line type and animal species differences. Unlike mouse YAMC and human Caco2/bbe cells, IPEC-J2 is a unique nontumorigenic, nontransformed porcine jejunum cell line (Ren et al. 2001; Petrof et al. 2004; Brosnahan and Brown 2012). This may also have contributed to the unexpected finding that the LGG strain and its supernatant were not able to induce HSP27 expression significantly in our study, whereas a solid effect was demonstrated previously in a YAMC cell line for the same strain of *Lactobacillus* and the same preparation procedure (Tao et al. 2006). Instead, we found that *L. johnsonii* and *L. reuteri* and its supernatant increased HSP27 expression in IPEC-J2 cells. The overall high HSP27 expression and strong induction by *L. johnsonii* and *L. reuteri* suggest that the porcine mucosa may establish a strategy to prepare and react to various stimuli immediately following their contact with IECs. HSP27 can bind directly to cytoskeleton protein F-actin and stabilize the TJ complex (Mounier and Arrigo 2002). Interestingly, ZO-1 can also bind directly to F-actin to regulate cytoskeleton organization (Ulluwishewa et al. 2011). Although further studies are necessary to identify interactions between HSPs and TJ proteins and the molecular pathways involved, it is tempting to speculate that lactobacilli, or their metabolites, would induce cytoprotective HSP27 expression, interacting with ZO-1 through F-actin that would regulate transmembrane TJ protein distribution and consequently strengthen the gut barrier function.

Unlike the overall strong induction of HSP27, the expression of HSP72 was only induced by the supernatant of *L. reuteri* in our study, indicating that the effect on HSP72 may be more stimulus-specific. HSP72 exerts chaperone activity that rescues intracellular proteins from irreversible denaturation and restores damaged epithelium (Borges et al. 2012). There is evidence that induction of HSP72 plays a major role in cytoprotection against intestinal damage, such as oxidative stress, dextran sodium sulfate-induced colitis, and human inflammatory bowel disease, compared with small HSPs such as HSP25/27 (Musch et al. 1999; Petrof et al. 2004; Hu et al. 2009; Liedel et al. 2011). Previous studies have reported varying response times of probiotic treatment for HSP induction. The probiotic product VSL#3-induced HSP expression in YAMC cells at 6 h (Petrof et al. 2004). In a study on LGG supernatant, the induction of HSP25 took 18 h to appear, whereas HSP72 expression began after 4 h (Tao et al. 2006). Furthermore, 16-h incubation was necessary for heat-killed *Lactobacillus brevis* to induce HSPs in Caco-2/bbe cells (Ueno et al. 2011). All together, these results indicate that the period needed for HSP induction differs between HSP27 and HSP72, and also varies depending on whether bacteria or supernatant are used.

One of the objectives of the present study was to investigate whether bacterial supernatant can achieve the same effect as live bacteria. We observed that live LGG treatment caused an IPEC-J2 cell viability reduction within a 12-h incubation period. The supernatant of overnight bacterial cultures carries putative bioactive compounds, for example, short chain fatty acids, secreted proteins, and peptides, accompanied by an acidic pH. We suspect that the low pH in the LGG incubation (pH 4.00) can be attributed at least partly to varied experimental effects, including cell viability, since the normal microclimate in pig small intestine lumen (Wenk 2001) and in IPEC-J2 cell culture medium is about pH 7.4.

All bacteria, including ETEC, were able to attach to the IPEC-J2 monolayer with 2-h incubation in this study. These results are in agreement with those reported in other studies and show that IPEC-J2 cell line is functionally valid for ETEC infection studies (Koh et al. 2008; Duan et al. 2012; Klingspor et al. 2014). Furthermore, *L. johnsonii* and LGG exhibited competitive advantages over *L. reuteri* with regard to ETEC inhibition. The ability of lactobacilli to adhere to IECs might not only promote pathogen removal (Roselli et al. 2007), but also prevent its own elimination by intestinal peristalsis in vivo (Granato et al. 1999). In the present study, the bacterial supernatant did not suppress ETEC adhesion. The effect observed here is more likely due to competition of lactobacilli with ETEC for binding sites in IECs. It is suggested that *Lactobacillus,* for instance *L. reuteri* R2LC, exhibits robust capacity to adhere to the intestinal barrier (Brosnahan and Brown 2012; Dicksved et al. 2012).

In conclusion, this study demonstrated that the conventional probiotic LGG and our own newly isolated porcine *L. johnsonii* and *L. reuteri* strains exert differing protective activities in IPEC-J2 cells. The strongest effect was seen with the *L. reuteri* strain, which provided substantial protection of the gut mucosa by preserving the TJ structure while being challenged with ETEC. The maintenance of barrier integrity was achieved at least partly through production of HSP27 and HSP72. Moreover, *L. johnsonii* and LGG were able to interact with ETEC directly and reduce its detrimental effect on IPEC-J2 cells, highlighting their probiotic potential and warranting more detailed characterization in future studies.
